# Expert consensus on the availability and consumption of herbal medicinal products in Thai pharmacies: An e-Delphi study

**DOI:** 10.1371/journal.pone.0360797

**Published:** 2026-10-15

**Authors:** Thanat Nakaphan, Monthaka Teerachaisakul, Chatchai Chaotham, Krit Pongpirul

**Affiliations:** 1 Department of Preventive and Social Medicine, Faculty of Medicine, Chulalongkorn University, Bangkok, Thailand; 2 Division of Herb for Economy, Department of Thai Traditional and Alternative Medicine, Ministry of Public Health, Nonthaburi, Thailand; 3 Department of Biochemistry and Microbiology, Faculty of Pharmaceutical Sciences, Chulalongkorn University, Bangkok, Thailand; 4 Center of Excellence in Preventive and Integrative Medicine (CE-PIM), Faculty of Medicine, Chulalongkorn University, Bangkok, Thailand; 5 Department of Infection Biology and Microbiomes, University of Liverpool, Liverpool, United Kingdom; 6 Bumrungrad International Hospital, Bangkok, Thailand; University of Ghana, GHANA

## Abstract

**Introduction:**

Despite the growing use of herbal medicinal products (HMPs) in Thailand, factors relevant to their availability and consumption in pharmacies remain insufficiently characterized. Pharmacies and healthcare professionals may play important roles in improving the availability and promoting their appropriate use.

**Methods:**

A three-round e-Delphi study was conducted among 30 multidisciplinary experts to identify and establish consensus on factors considered relevant to the availability and consumption of HMPs in pharmacies across Thailand. Experts rated the relevance and impact of each factor using a 7-point Likert scale. Consensus was defined as a Quality of the Lower Approximation (QL) score ≥ 0.75.

**Results:**

Of the 34 factors assessed, 25 reached consensus across four domains: (1) herbal medicinal products, (2) product information, (3) professional responsibilities, and (4) pharmacy operations. The National List of Essential Herbal Drugs received the highest consensus score (QL = 1.00). All nine product information factors reached consensus. Professional factors reaching consensus included product recommendation, knowledge of HMP categories, and knowledge of evidence-based efficacy and safety. Pharmacy-related factors reaching consensus included pharmacy type, business model, participation in national health programs, and location.

**Conclusion:**

Expert consensus identified a set of factors considered relevant to HMP availability and consumption in Thai pharmacies. These findings provided a framework for future empirical studies and may inform policies and interventions related to product information, professional competency, and pharmacy operations.

## Introduction

Herbal medicinal products (HMPs) are widely used in Thailand and constitute an important component of both traditional and contemporary healthcare. The Thai market for HMPs reached approximately 44.8 billion Baht in 2024, reflecting their substantial economic and healthcare relevance [1]. Nevertheless, access to HMPs may vary across healthcare settings and geographic areas. Retail pharmacies account for approximately 20% of pharmaceutical product distribution in Thailand, with the majority occurring through hospitals [2]. As accessible community-based healthcare facilities, pharmacies may therefore provide an important channel for improving access to HMPs [3].

Availability is an important dimension of access to medicines. The World Health Organization (WHO), in collaboration with Health Action International (HAI), developed a standardized framework for assessing the availability and affordability of essential medicines [4]. This framework and related approaches have been applied to both conventional medicines and HMPs [5,6]. In Thailand, the use of HMPs is influenced by multiple factors, including accessibility, affordability, perceived therapeutic benefits, geographic context, and individual characteristics [7,8]. Product quality, regulation, and regional differences may further affect their availability and use [9].

As key retail settings for both conventional and traditional medicines, pharmacies may play an important role in improving access to HMPs. In Thailand, pharmacies provide an important point of access to HMPs, although regional and socioeconomic differences may influence patterns of herbal medicine use [10]. Beyond accessibility, the quality and safety of HMPs remain important considerations because herbal products may vary in composition, standardization, and quality, underscoring the importance of appropriate regulatory oversight and quality assurance [11,12]. However, the factors considered relevant to HMP availability and consumption within pharmacy settings have not been systematically characterized and prioritized in Thailand.

Given this evidence gap, this study aimed to identify and establish consensus on factors considered relevant to the availability and consumption of HMPs in Thai pharmacies. We employed a three-round e-Delphi method to gather expert opinions from professionals with expertise in HMPs, pharmacy, public health, and retail management. The e-Delphi technique offers several advantages, including geographic reach, anonymity, and iterative feedback, minimizing bias while refining expert consensus on complex issues [13,14]. The resulting consensus may provide a framework for future empirical investigation and inform the development of policies and interventions related to HMP availability and consumption in Thai pharmacies.

## Methods

### Participants

Experts were selected based on predefined eligibility criteria reflecting relevant professional, academic, policy, or practice experience related to HMPs. To be eligible, participants were required to meet at least one of the following criteria:

1) Government expertise: Licensed pharmacists or medical professionals with expertise or specialization in policy or healthcare service units under the Ministry of Public Health.
2) Professional qualifications: Individuals with a doctoral degree in pharmacy or a related field, a valid pharmacy license, and certification in herbal pharmacy from the Thai College of Herbal Pharmacy, or individuals holding leadership positions in the Community Pharmacy Association of Thailand.
3) Academic expertise: Faculty members of recognized public or private universities specializing in pharmacy, medicine, or herbal sciences.
4) Traditional and integrative medicine: Experts with a doctoral degree and valid licenses in traditional or integrative medicine, affiliated with academic institutions specializing in traditional medicine or integrative health.
5) Professional experience: Licensed pharmacists or other healthcare professionals with at least five years of experience managing pharmacies or businesses selling conventional medicines, traditional medicines, or HMPs.

All participants were informed of their right to withdraw from the study at any time without consequence. All three rounds of the e-Delphi questionnaire were administered electronically using Google Forms. Electronic informed consent was obtained from all participants before participation. Participants documented their voluntary agreement to participate by checking a consent box before proceeding to the questionnaire. The study was approved by the Institutional Review Board of the Faculty of Medicine, Chulalongkorn University, Bangkok, Thailand (approval no. 0997/2023).

### e-Delphi exploration

A three-round e-Delphi study was conducted between 1 September 2023 and 29 February 2024 to identify and establish expert consensus on factors considered relevant to the availability and consumption of HMPs in Thai pharmacies (Fig 1). In this study, the term “pharmacies” encompasses both modern pharmacies and traditional medicine pharmacies operating under the relevant Thai licensing framework.

**Fig 1:**
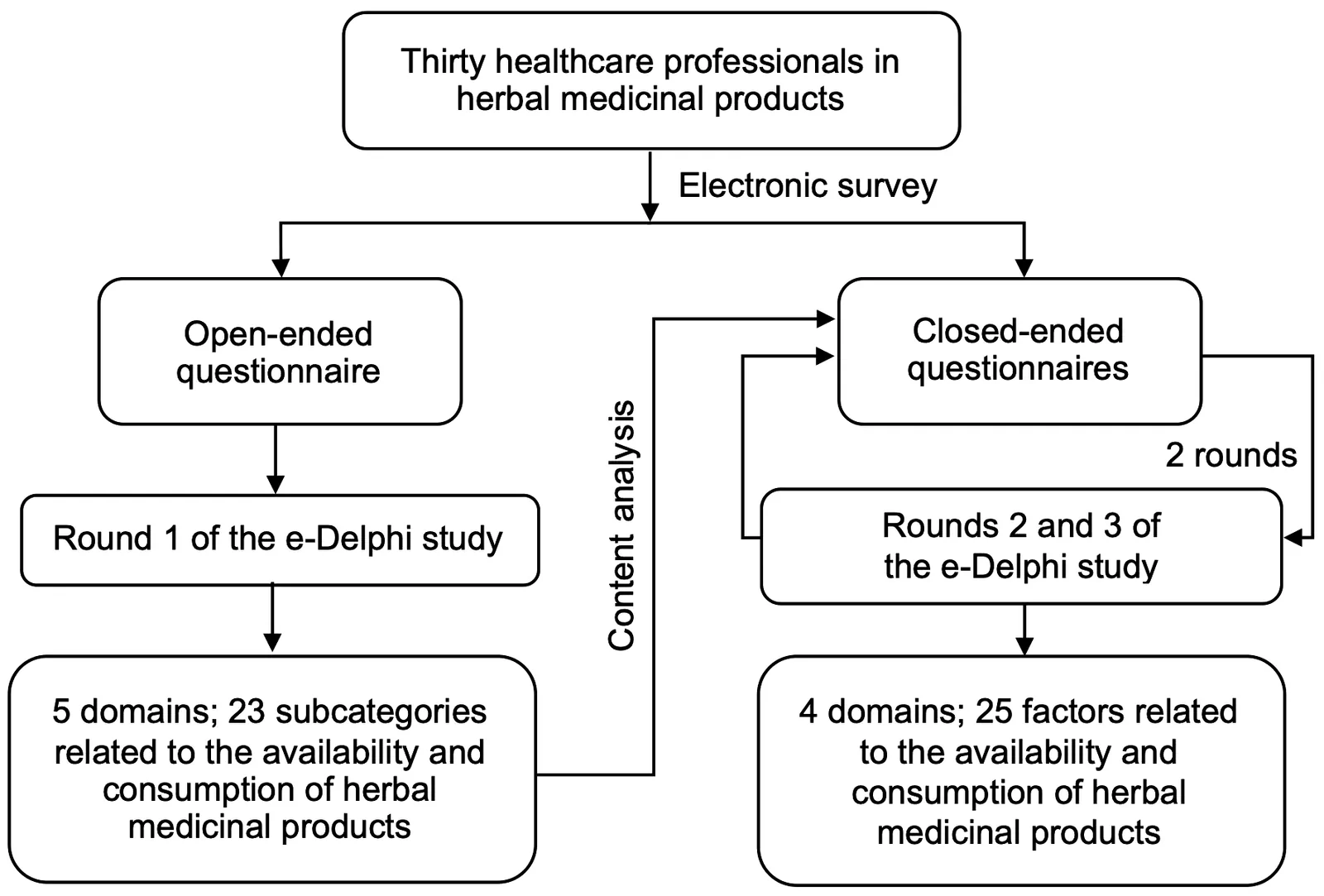
Overview of the three-round e-Delphi process. The process involved 30 experts and comprised an initial open-ended questionnaire (Round 1), followed by content analysis and two rounds of closed-ended questionnaires (Rounds 2 and 3), resulting in expert consensus on 25 factors across four domains.

Round 1 (Qualitative exploration): Experts completed an open-ended questionnaire to propose factors considered relevant to HMP availability and consumption. They were given two weeks to submit their responses.

Round 2 (Quantitative rating): Responses from Round 1 underwent content analysis and were used to develop a structured questionnaire [15]. Experts rated the relevance (a1) and impact (a2) of each factor using a 7-point Likert scale (1 = lowest, 7 = highest). Experts were given one week to complete this round.

Round 3 (Consensus refinement): A summary of the Round 2 responses was provided to the experts, who were asked to reassess their ratings in light of the group feedback. Factors with a Quality of the Lower Approximation (QL) score ≥ 0.75 were considered to have reached consensus, indicating no substantial disagreement among experts [16].

### Data analysis

Descriptive statistics were used to summarize participant characteristics and expert ratings of factors considered relevant to HMP availability and consumption. Means and standard deviations were calculated for the relevance and impact ratings of each factor in Rounds 2 and 3.

Consensus definition: QL values were calculated separately for Rounds 2 and 3 as the proportion of experts who rated both relevance (a1) and impact (a2) ≥ 5 on the 7-point Likert scale. A QL value ≥ 0.75 was predefined as the threshold for consensus, indicating agreement among at least 75% of experts. Final consensus status was determined based on the Round 3 QL values.

Data interpretation: Factors that reached consensus in Round 3 were categorized into four domains: (1) Herbal medicinal products, (2) Product information, (3) Professional responsibilities, and (4) Pharmacy operations.

## Results

### Expert demographics

A total of 30 experts participated in all three rounds of the e-Delphi study (Table 1; S1 Data). The majority were male (66.67%), and the panel included 22 pharmacists, 5 Thai traditional doctors, and 3 medical doctors. Participants represented four professional sectors: academic institutions (n = 14), government agencies (n = 7), hospitals (n = 3), and pharmacies (n = 6). All five Thai traditional doctors were affiliated with pharmacies.

**Table 1:** Characteristics of participants in the e-Delphi study (n = 30).

Characteristic	Number of participants, n
**Gender** Male Female	20 10
**Age (years)** 30–40 41–50 51–60 >60	10 12 4 4
**Educational degree** Bachelor’s degree Master’s degree Doctoral degree	4 7 19
**Profession** Pharmacist Medical doctor Thai traditional doctor	22 3 5
**Professional sector** Government agency Hospital Pharmacy Academic institution (university)	7 3 6 14
**Years of professional experience** 5-10 years >10 years	9 21
**Geographic region** Bangkok Central North Northeast South East West	8 10 2 2 4 1 3

Although the minimum eligibility criterion was five years of professional experience, the majority of participants (70%; n = 21) had more than ten years of experience in their respective fields. Geographically, participants represented all regions of Thailand, with the largest proportions from the Central region (33.33%) and Bangkok (26.67%). The panel included experts from multiple professional sectors and geographic regions, providing diverse perspectives relevant to HMP availability and consumption in Thailand.

### Opinions obtained from the open-ended e-Delphi round

The first round of the e-Delphi study involved seven open-ended questions (S2 Data) designed to elicit expert opinions on factors considered relevant to the availability and consumption of HMPs in Thai pharmacies. The questions addressed four broad areas: (1) characteristics of HMPs, (2) pharmacy operations, (3) roles and responsibilities of healthcare professionals, and (4) mechanisms for facilitating HMP use.

A suggestion box was also provided to allow experts to propose additional factors not explicitly addressed by the questions. The qualitative responses were analyzed and categorized, and the resulting themes and illustrative quotations are summarized in Table 2.

**Table 2:** Themes identified from expert responses to the Round 1 open-ended questionnaire on factors considered relevant to the availability and consumption of herbal medicinal products (HMPs) in Thai pharmacies.

Theme / Subcategory	Illustrative quotations
**1. Herbal medicinal products** 1.1 National List of Essential Herbal Drugs 1.2 Herbal medicines recommended as first-line treatment 1.3 Traditional household remedies 1.4 Traditional herbal formulas 1.5 Herbal medicines used in public health facilities 1.6 Herbal Champions under the National Herbal Action Plan 1.7 Single or combined products 1.8 Route of administration 1.9 Treatment category	*“The types of diseases or conditions that the public commonly seeks to self-medicate with, such as during the COVID-19 pandemic”* *“The National List of Essential Medicines, as well as those outside the list, supported by credible empirical evidence”* *“Thirty-five herbal medicines are included in the primary healthcare unit under the National Health Security Office (NHSO) because they are actively promoted for implementation within the pharmacy setting”*
**2. Pharmacies** 2.1 Category of pharmacy (e.g., modern, traditional) 2.2 Business model 2.3 Accreditation by the Office of Community Pharmacy Accreditation 2.4 Participation in National Health Security Office (NHSO) programs 2.5 Service area 2.6 Regional location 2.7 Nearby location	*“Stand-alone and certified pharmacies are more likely to dispense herbal medicinal products compared to chain pharmacies, which tend to prefer dispensing herbal supplement products”* *“Certified stand-alone or chain pharmacies, along with their locations (e.g., urban areas, shopping malls, communities, and rural regions).”* *“Consultation service areas for the use of herbal medicines provided by pharmacists”*
**3. Professionals on duty** 3.1 Product recommendation 3.2 Knowledge of herbal medicinal products 3.3 Healthcare services	*“Knowledge, experience, and competencies are essential for the rational and safe use of herbal medicines, aiming to enhance the quality of customer counseling.”* *“With or without telemedicine services”*
**4. Facilitating HMP use** 4.1 Accessibility of uncommon herbal medicines 4.2 Manufacturing and supply chain 4.3 Product information	*“Brand names are important as they can be associated with reliable sources of production or distribution.”* *“Stock shortages from manufacturers are due to seasonal availability and low demand.”* *“The pricing mechanisms and the prices expected by consumers.”*
**5. Other Suggestions** 5.1 Manufacturers and suppliers (location, quality, service, promotion)	*“The sources of herbal medicinal products, including direct procurement from manufacturers, intermediaries, or in-house production”* *“The manufacturing standards, such as Good Agricultural Practices (GAP) and Good Manufacturing Practices (GMP)”*

Note: Traditional household remedies are classified according to their regulatory status for household use, whereas first-line herbal medicines are recommended as initial treatment options for specified conditions. Traditional herbal formulas are defined according to their basis in traditional medicine knowledge, whereas herbal medicines used in public health facilities are categorized according to their use within the public healthcare system. These categories therefore represent different, potentially overlapping criteria for classifying or selecting HMPs.

Several experts highlighted the regional location of pharmacies and the quality of services provided by product suppliers as potentially relevant to HMP availability and consumption. These and other responses informed the development of the factors subsequently evaluated in the closed-ended rounds.

### Consensus on availability and consumption factors

The 23 subcategories identified from the open-ended responses in Round 1 were refined and expanded into 34 specific factors, which were organized into four domains for the closed-ended questionnaire: (1) Herbal medicinal products, (2) Product information, (3) Professionals, and (4) Pharmacy operations. In Round 2, experts rated each factor on two attributes, relevance (a1) and impact (a2), using a 7-point Likert scale. In Round 3, experts received a summary of the Round 2 results and were given the opportunity to reassess their ratings in light of the group feedback.

A factor was considered to have reached consensus if its Quality of the Lower Approximation (QL) score was ≥ 0.75. Final consensus status was determined based on the Round 3 QL values. QL values and consensus status for Rounds 2 and 3 are presented in Tables 3–6.

**Table 3:** Quality of the Lower Approximation (QL) values for herbal medicinal product factors considered relevant to HMP availability and consumption in Thai pharmacies.

Code	Factor	Round 2 QL	Round 2 decision	Round 3 QL	Round 3 decision
1.1	Widely used products	0.90	Consensus	0.87	Consensus
1.2	High demand or customer-requested products	0.73	Non-consensus	0.90	Consensus
1.3	Products with evidence of potential health risks	0.63	Non-consensus	0.57	Non-consensus
1.4	Substitutes for conventional medicines	0.67	Non-consensus	0.70	Non-consensus
1.5	Herbal medicines included for use in pharmacy-based primary healthcare services under the National Health Security Office (NHSO) program	0.80	Consensus	0.83	Consensus
1.6	National List of Essential Herbal Drugs established by the National Drug System Committee	1.00	Consensus	1.00	Consensus
1.7	Traditional household remedy products listed by the Ministry of Public Health	0.60	Non-consensus	0.63	Non-consensus
1.8	Herbal Champions under the National Herbal Action Plan	0.63	Non-consensus	0.73	Non-consensus
1.9	Products with national reference prices (per National Drug System Development Committee)	0.53	Non-consensus	0.53	Non-consensus
1.10	Single or combination products	0.67	Non-consensus	0.93	Consensus
1.11	Products categorized by physiological system (e.g., respiratory, GI tract)	0.57	Non-consensus	0.70	Non-consensus
1.12	Products categorized by symptom or indication (e.g., carminative, antiflatulent)	0.83	Consensus	0.83	Consensus
1.13	Route of administration (e.g., internal vs. external use)	0.73	Non-consensus	0.93	Consensus

Note: QL, Quality of the Lower Approximation. Consensus was defined as QL ≥ 0.75. Final consensus status was determined based on Round 3 QL values.

**Table 4:** Quality of the Lower Approximation (QL) values for product information factors considered relevant to HMP availability and consumption in Thai pharmacies.

Code	Factor	Round 2 QL	Round 2 decision	Round 3 QL	Round 3 decision
2.1	Medication information leaflet (as per Thai FDA guidelines)	0.87	Consensus	0.97	Consensus
2.2	Generic name	0.87	Consensus	0.97	Consensus
2.3	Trade name	0.67	Non-consensus	0.80	Consensus
2.4	Dosage form	0.83	Consensus	0.93	Consensus
2.5	Strength	0.83	Consensus	0.83	Consensus
2.6	Registration number	0.87	Consensus	1.00	Consensus
2.7	Unit price	0.63	Non-consensus	0.80	Consensus
2.8	Manufacturer	0.83	Consensus	0.93	Consensus
2.9	Supplier (location, quality, service, and promotion)	0.67	Non-consensus	0.87	Consensus

Note: QL, Quality of the Lower Approximation. Consensus was defined as QL ≥ 0.75. Final consensus status was determined based on Round 3 QL values.

**Table 5:** Quality of the Lower Approximation (QL) values for professional factors considered relevant to HMP availability and consumption in Thai pharmacies.

Code	Factor	Round 2 QL	Round 2 decision	Round 3 QL	Round 3 decision
3.1	Product recommendation	0.70	Non-consensus	0.83	Consensus
3.2	Knowledge of HMP categories	0.90	Consensus	0.93	Consensus
3.3	Knowledge of evidence-based efficacy and safety	0.73	Non-consensus	0.83	Consensus
3.4	Telepharmacy and home delivery services	0.53	Non-consensus	0.70	Non-consensus

Note: QL, Quality of the Lower Approximation. Consensus was defined as QL ≥ 0.75. Final consensus status was determined based on Round 3 QL values.

**Table 6:** Quality of the Lower Approximation (QL) values for pharmacy-related factors considered relevant to HMP availability and consumption in Thai pharmacies.

Code	Factor	Round 2 QL	Round 2 decision	Round 3 QL	Round 3 decision
4.1	Category of pharmacy (e.g., modern, traditional, herbal product store)	0.83	Consensus	0.93	Consensus
4.2	Business model (e.g., stand-alone vs. chain pharmacy)	0.87	Consensus	0.90	Consensus
4.3	Retail or wholesale status	0.73	Non-consensus	0.87	Consensus
4.4	Pharmacy floor area	0.67	Non-consensus	0.70	Non-consensus
4.5	Consultation area within the store	0.53	Non-consensus	0.57	Non-consensus
4.6	Participation in national health promotion programs (e.g., Community Pharmacy Project, Near Home Medicine Pickup)	0.77	Consensus	0.87	Consensus
4.7	Nearby location (e.g., proximity to hospital, community, or clinic)	0.83	Consensus	0.90	Consensus
4.8	Regional location (e.g., Bangkok, Central Region)	0.83	Consensus	0.90	Consensus

Note: QL, Quality of the Lower Approximation. Consensus was defined as QL ≥ 0.75. Final consensus status was determined based on Round 3 QL values.

Of the 34 factors assessed, 25 reached consensus, while 9 did not reach the consensus threshold in Round 3. The factor “National List of Essential Herbal Drugs” (1.6) received the highest QL score of 1.00, indicating all experts rated both its relevance and impact at or above the predefined threshold. In contrast, “Products with national reference prices” (1.9) received the lowest QL score of 0.53 and did not reach consensus. All nine factors in Domain 2: Product information reached consensus by Round 3 (Table 4), indicating strong expert agreement on the relevance of product information to HMP availability and consumption in Thai pharmacies.

Fig 2 compares the QL values obtained in Rounds 2 and 3 of the e-Delphi process. QL values increased for several factors, including factors related to product information, professional responsibilities, and pharmacy operations, reflecting increased expert agreement following the Round 2 feedback.

**Fig 2:**
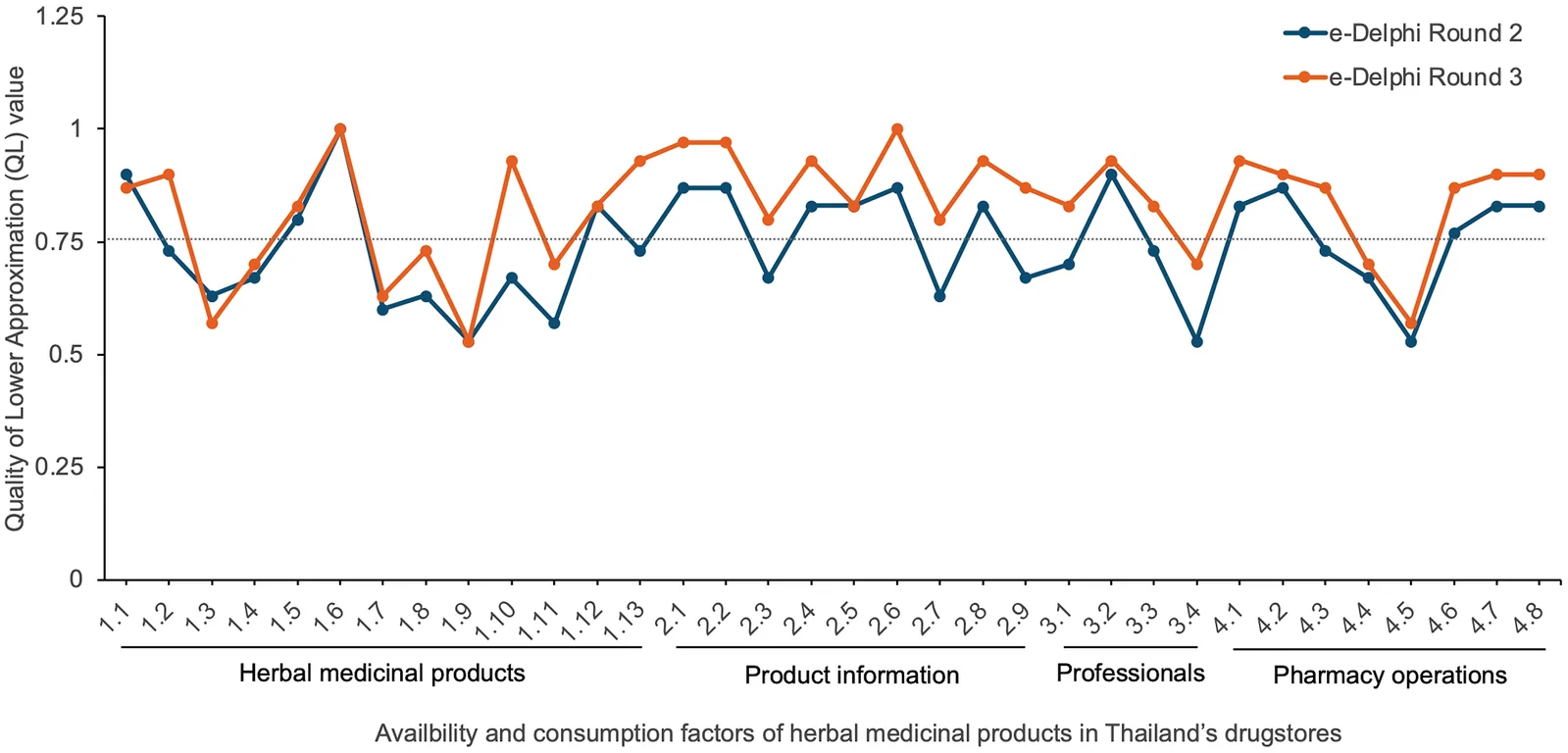
Consensus progression across e-Delphi Rounds 2 and 3. Quality of the Lower Approximation (QL) values for factors considered relevant to the availability and consumption of herbal medicinal products in Thai pharmacies are shown for the two closed-ended e-Delphi rounds. The predefined threshold for consensus was QL ≥ 0.75.

## Discussion

Herbal medicinal products (HMPs) continue to play an important role in healthcare systems, particularly in low- and middle-income countries (LMICs), where traditional medicines remain widely used [17]. Despite their widespread use, integration of HMPs into formal health systems faces challenges related to quality control, standardization, safety, efficacy, and accessibility. In Thailand, herbal medicines constitute approximately 10% of the National List of Essential Medicines [9]. Standardization of traditional formulations and development of reliable dosage forms may contribute to product quality, safety, and confidence in their use [18,19].

Access to essential medicines is a fundamental component of healthcare delivery, yet disparities in medicine availability remain substantial. Many developing countries continue to experience in equitable access to essential medicines [20,21]. Globally, approximately 15% of the population consumes 90% of pharmaceutical products [22]. Within LMICs, barriers including limited health literacy, incomplete prescription fulfillment, and geographic isolation may further restrict access [23,24]. Community pharmacies may help address some of these barriers because of their accessibility within communities. In Thailand, more than 20,000 licensed pharmacies operate under the Thai FDA, with approximately 70% located outside Bangkok [25]. Previous research has also shown that demographic characteristics, consumer attitudes, and media exposure are associated with HMP utilization in Thailand [26].

To our knowledge, this is the first e-Delphi study to establish expert consensus on factors considered relevant to HMP availability and consumption in Thai pharmacies. Among the HMP-related factors, the National List of Essential Herbal Drugs and herbal medicines included for use in pharmacy-based primary healthcare services under the National Health Security Office (NHSO) program reached consensus (Table 3), suggesting that experts considered national policy and healthcare programs relevant to HMP availability and consumption. Conversely, several factors, including products with evidence of potential health risks, substitutes for conventional medicines, traditional household remedies, and Herbal Champions under the National Herbal Action Plan, did not reach the predefined consensus threshold. These findings indicate differences in expert agreement regarding the relevance and impact of different HMP classification and policy-related categories and warrant further empirical investigation.

Professional knowledge and responsibilities also emerged as an important area of expert agreement (Table 5). Consensus was reached for product recommendation, knowledge of HMP categories, and knowledge of evidence-based efficacy and safety. This is consistent with previous studies indicating that healthcare professionals recognize their responsibility to advise consumers about HMPs but may lack sufficient training or confidence [27]. In China, where Traditional Chinese Medicine is integrated into the healthcare system, pharmacists also contribute to managing HMP-related risks, including herb-drug interactions [28]. More broadly, pharmacists may contribute throughout the medicine management process, including procurement, quality assurance, dispensing, and consumer counseling [29]. While the present survey addressed knowledge of efficacy and safety broadly, specific professional competencies, including knowledge of potential adverse effects, contraindications, precautions, and proper storage conditions, were not assessed as separate factors. The ability to provide clear instructions on appropriate product use and timing should also be considered in future studies.

Although telepharmacy and home delivery services have demonstrated potential to improve access to healthcare services [30], this factor did not reach the predefined consensus threshold in the present study. The reasons for this finding were not directly examined and therefore cannot be determined from the Delphi results. Future research could examine the acceptability, cost-effectiveness, safety, and impact of telepharmacy models on HMP delivery in community pharmacy settings [31].

Product information showed particularly consistent expert agreement, with all nine factors in this domain reaching consensus by Round 3 (Table 4). Medication information leaflets may support the safe and appropriate use of medicines [32], although concerns have been raised regarding readability, technical complexity, and their potential effects on patients [33]. Previous research has also identified deficiencies in information concerning contraindications and interactions [34]. In the present study, consensus was reached on medication information leaflets, generic and trade names, dosage form, strength, registration number, unit price, manufacturer, and supplier information, indicating strong expert agreement on the relevance of accessible and standardized product information to HMP availability and consumption. Future studies should also examine additional product information, such as dosage, active ingredients, cautions, batch number, shelf life, and storage conditions, which were not assessed as separate factors in the closed-ended e-Delphi survey.

International evidence provides additional context for the pharmacy-related findings. In São Paulo, Brazil, only 11% of primary care facilities offered herbal medicines, with variation according to socioeconomic and demographic characteristics [35]. Similarly, a Thai study found that 28.6% of Bangkok residents reported using HMPs from the National List of Essential Medicines, with utilization varying according to socioeconomic and demographic characteristics [26]. In the present study, consensus was reached for several pharmacy-related factors, including pharmacy category, business model, retail or wholesale status, participation in national health promotion programs, nearby location, and regional location. These findings suggest that experts considered both organizational and geographic characteristics of pharmacies relevant to HMP availability and consumption. Previous research has likewise indicated that healthcare financing, cultural preferences, and beliefs may influence patterns of traditional medicine use [36,37].

Inventory-related issues also emerged during the open-ended round and were considered within the broader themes of product availability, procurement, and supply chain. However, inventory control methods were not independently assessed in the closed-ended e-Delphi survey and should be further examined as a distinct operational factor in future studies.

The e-Delphi method enabled structured elicitation and refinement of expert opinions on a complex and under-explored issue. Its strengths include geographically diverse participation, anonymity and iterative feedback, which can reduce the influence of dominant individuals and facilitate structured refinement of expert judgments [38]. Nevertheless, the method has limitations, including potential ambiguity in interpreting rating scales, participant fatigue, and challenges in achieving comprehensive stakeholder representation [39]. Although the panel included experts from multiple professions, sectors, and geographic regions, the findings represent expert consensus rather than population-level estimates or empirical evidence of causal relationships. A larger and more diverse panel, including stakeholders such as over-the-counter medicine sellers, could provide additional perspectives.

The Quality of the Lower Approximation (QL) metric was applied separately in Rounds 2 and 3 to evaluate expert consensus. Final consensus status was determined from the Round 3 QL values, following the provision of Round 2 group feedback and reassessment by the experts. QL values increased for several factors between Rounds 2 and 3, although some remained unchanged or decreased. This pattern illustrates how iterative feedback allowed participants to reconsider their judgments without necessarily producing convergence for every factor [40]. The robustness of Delphi consensus also depends on clear criteria, appropriate panel composition, and transparent iteration [41]. Complementary descriptive statistics, including means and standard deviations, provide additional information on the distribution of expert ratings [42]. These statistics were calculated separately for relevance and impact ratings in Rounds 2 and 3 and are reported in S3 Data, while consensus was determined using the predefined QL criterion.

## Conclusion

This study identified and established consensus on factors considered relevant to the availability and consumption of HMPs in Thai pharmacies. Of the 34 factors evaluated, 25 reached the predefined consensus threshold in Round 3 across four domains: herbal medicinal products, product information, professional responsibilities, and pharmacy operations. Particularly strong consensus was observed for the National List of Essential Herbal Drugs and product information factors, while professional knowledge and several organizational and geographic characteristics of pharmacies also reached consensus.

The identified factors provide a framework for future empirical studies on HMP availability and consumption in Thai pharmacies and may inform the development of pharmacy, regulatory, and professional interventions. Further research is needed to determine the relative importance of these factors and their associations with actual HMP availability and consumption.

## Supporting information

S1 Data De-identified characteristics of participants in the e-Delphi study. (XLSX)

S2 Data Round 1 open-ended questionnaire, analytical mapping of responses, and de-identified original responses. (XLSX)

S3 Data Rounds 2 and 3 closed-ended questionnaires, de-identified individual ratings of relevance and impact, and corresponding descriptive statistics. (XLSX)
