# Supplementary material for: Expert consensus on the availability and consumption of herbal medicinal products in Thai pharmacies: An e-Delphi study
Source: PLoS One. 2026 Oct 15;21(10):e0360797. doi: 10.1371/journal.pone.0360797 (PMC13671551; doi:10.1371/journal.pone.0360797)
Supplement: Supplementary file 1 [file pone.0360797.s004.docx]

Review Comments on PONE-D-25-54073

The manuscript describes a very important topic that is both relevant and timely as the world shifts gradually back towards natural remedies for disease prevention, treatment and for general wellbeing of the human population. Finding from studies such as this can inform the formulation and rollout of policies to better regulate herbal medicine practice worldwide.

There are however some issues with the manuscript that may require the attention of the authors.

1. It would have been great to include over the counter medicine sellers (OTCMS) in the study as respondents, since they are the main distributors and dispensers of herbal medicines in most countries.
2. Given the importance of the study, a relatively higher number of respondents from a wider professional background would have been great.
3. The there is indication that the questionnaire were administered electronically but the specific software or platform used such google forms etc. was not mentioned
4. In lines 56, 57, and 63, the abbreviations HPMS and HMPS have used interchangeably. Do they represent the same thing?
5. In line 138, Table 1, n=3, however the total respondents in roll 4 is 31 and not 30. Can the authors clarify this difference.
6. In lines 35-37, the 30 participants represented 37 professional sectors. Can the authors clarify whether or not some participants represented more than one professional sector. It is also important to know which professional sector (academic institutions, hospitals government agencies and community-based pharmacies) that was represented by the 5 Thai Traditional Doctors.
7. In line 136, the number of academic institutions is given as 21 and in Table 1, roll 5, the number of academic institutions is given as 14. The authors should which of the two figures is correct.
8. Line 169: The authors should revise the sentence.
