# Supplementary material for: Expert consensus on the availability and consumption of herbal medicinal products in Thai pharmacies: An e-Delphi study
Source: PLoS One. 2026 Oct 15;21(10):e0360797. doi: 10.1371/journal.pone.0360797 (PMC13671551; doi:10.1371/journal.pone.0360797)
Supplement: Peer review history

# Peer review: Decision Letter 0

**DOI:** 10.1371/journal.pone.0360797.r001

**Authors:** Ofosua Adi-Dako

27 Jul 2026

Dear Dr. Krit Pongpirul,

plosone@plos.org. When you're ready to submit your revision, log on to https://www.editorialmanager.com/pone/ and select the 'Submissions Needing Revision' folder to locate your manuscript file.

- A letter that responds to each point raised by the academic editor and reviewer(s). You should upload this letter as a separate file labeled 'Response to Reviewers'.
- A marked-up copy of your manuscript that highlights changes made to the original version. You should upload this as a separate file labeled 'Revised Manuscript with Track Changes'.
- An unmarked version of your revised paper without tracked changes. You should upload this as a separate file labeled 'Manuscript'.

If you would like to make changes to your financial disclosure, please include your updated statement in your cover letter. Guidelines for resubmitting your figure files are available below the reviewer comments at the end of this letter.

If applicable, we recommend that you deposit your laboratory protocols in protocols.io to enhance the reproducibility of your results. Protocols.io assigns your protocol its own identifier (DOI) so that it can be cited independently in the future. For instructions see: https://journals.plos.org/plosone/s/submission-guidelines#loc-laboratory-protocols. Additionally, PLOS ONE offers an option for publishing peer-reviewed Lab Protocol articles, which describe protocols hosted on protocols.io. Read more information on sharing protocols at https://plos.org/protocols?utm_medium=editorial-email&utm_source=authorletters&utm_campaign=protocols.

As the corresponding author, your ORCID iD is verified in the submission system and will appear in the published article. PLOS supports the use of ORCID, and we encourage all coauthors to register for an ORCID iD and use it as well. Please encourage your coauthors to verify their ORCID iD within the submission system before final acceptance, as unverified ORCID iDs will not appear in the published article. *Only*  the individual author can complete the verification step; PLOS staff *cannot*  verify ORCID iDs on behalf of authors.

We look forward to receiving your revised manuscript.

Kind regards,

Ofosua Adi-Dako

Academic Editor

PLOS One

Journal Requirements:

When submitting your revision, we need you to address these additional requirements.

1. Please ensure that your manuscript meets PLOS ONE's style requirements, including those for file naming. The PLOS ONE style templates can be found at

https://journals.plos.org/plosone/s/file?id=wjVg/PLOSOne_formatting_sample_main_body.pdf and

https://journals.plos.org/plosone/s/file?id=ba62/PLOSOne_formatting_sample_title_authors_affiliations.pdf

2. Please provide additional details regarding participant consent. In the ethics statement in the Methods and online submission information, please ensure that you have specified what type you obtained (for instance, written or verbal, and if verbal, how it was documented and witnessed). If your study included minors, state whether you obtained consent from parents or guardians. If the need for consent was waived by the ethics committee, please include this information.

Once you have amended this/these statement(s) in the Methods section of the manuscript, please add the same text to the “Ethics Statement” field of the submission form (via “Edit Submission”).

For additional information about PLOS ONE ethical requirements for human subjects research, please refer to http://journals.plos.org/plosone/s/submission-guidelines#loc-human-subjects-research.

3. Thank you for stating the following financial disclosure:

“This study was funded by the Agricultural Research Development Agency (Public Organization), Thailand (Grant No. PRP6505032160).”

Please state what role the funders took in the study. If the funders had no role, please state: "The funders had no role in study design, data collection and analysis, decision to publish, or preparation of the manuscript."

If this statement is not correct you must amend it as needed.

Please include this amended Role of Funder statement in your cover letter; we will change the online submission form on your behalf.

4. Thank you for stating the following in the Acknowledgments Section of your manuscript:

“TN gratefully acknowledges support from the Royal Golden Jubilee Ph.D. Program (RGJ-Ph.D. Program) under the National Research Council of Thailand, Ministry of Higher Education, Science, Research and Innovation (Grant No. NRCT5-RGJ63001-006).**”**

We note that you have provided funding information that is currently declared in your Funding Statement. However, funding information should not appear in the Acknowledgments section or other areas of your manuscript. We will only publish funding information present in the Funding Statement section of the online submission form.

Please remove any funding-related text from the manuscript and let us know how you would like to update your Funding Statement. Currently, your Funding Statement reads as follows:

“This study was funded by the Agricultural Research Development Agency (Public Organization), Thailand (Grant No. PRP6505032160).”

Please include your amended statements within your cover letter; we will change the online submission form on your behalf.

5. We note that your Data Availability Statement is currently as follows: All relevant data are within the manuscript and its Supporting Information files.

Please confirm at this time whether or not your submission contains all raw data required to replicate the results of your study. Authors must share the “minimal data set” for their submission. PLOS defines the minimal data set to consist of the data required to replicate all study findings reported in the article, as well as related metadata and methods (https://journals.plos.org/plosone/s/data-availability#loc-minimal-data-set-definition).

For example, authors should submit the following data:

- The values behind the means, standard deviations and other measures reported;

- The values used to build graphs;

- The points extracted from images for analysis.

Authors do not need to submit their entire data set if only a portion of the data was used in the reported study.

If your submission does not contain these data, please either upload them as Supporting Information files or deposit them to a stable, public repository and provide us with the relevant URLs, DOIs, or accession numbers. For a list of recommended repositories, please see https://journals.plos.org/plosone/s/recommended-repositories.

If there are ethical or legal restrictions on sharing a de-identified data set, please explain them in detail (e.g., data contain potentially sensitive information, data are owned by a third-party organization, etc.) and who has imposed them (e.g., an ethics committee). Please also provide contact information for a data access committee, ethics committee, or other institutional body to which data requests may be sent. If data are owned by a third party, please indicate how others may request data access.

6. If the reviewer comments include a recommendation to cite specific previously published works, please review and evaluate these publications to determine whether they are relevant and should be cited. There is no requirement to cite these works unless the editor has indicated otherwise.

7. Please review your reference list to ensure that it is complete and correct. If you have cited papers that have been retracted, please include the rationale for doing so in the manuscript text, or remove these references and replace them with relevant current references. Any changes to the reference list should be mentioned in the rebuttal letter that accompanies your revised manuscript. If you need to cite a retracted article, indicate the article’s retracted status in the References list and also include a citation and full reference for the retraction notice.

[Note: HTML markup is below. Please do not edit.]

Reviewers' comments:

Reviewer's Responses to Questions

**Comments to the Author**

1. Is the manuscript technically sound, and do the data support the conclusions?

Reviewer #1: Yes

Reviewer #2: No

Reviewer #3: Yes

**********

2. Has the statistical analysis been performed appropriately and rigorously?

Reviewer #1: N/A

Reviewer #2: No

Reviewer #3: Yes

**********

3. Have the authors made all data underlying the findings in their manuscript fully available?

The PLOS Data policy

Reviewer #1: Yes

Reviewer #2: No

Reviewer #3: Yes

**********

4. Is the manuscript presented in an intelligible fashion and written in standard English?

Reviewer #1: Yes

Reviewer #2: Yes

Reviewer #3: Yes

**********

Reviewer #1: The manuscript describes a very important topic that is both relevant and timely as the world shifts gradually back towards natural remedies for disease prevention, treatment and for general wellbeing of the human population. Finding from studies such as this can inform the formulation and rollout of policies to better regulate herbal medicine practice worldwide.

There are however some issues with the manuscript that may require the attention of the authors.

1. It would have been great to include over the counter medicine sellers (OTCMS) in the study as respondents, since they are the main distributors and dispensers of herbal medicines in most countries.

2. Given the importance of the study, a relatively higher number of respondents from a wider professional background would have been great.

3. The there is indication that the questionnaire were administered electronically but the specific software or platform used such google forms etc. was not mentioned

4. In lines 56, 57, and 63, the abbreviations HPMS and HMPS have used interchangeably. Do they represent the same thing?

5. In line 138, Table 1, n=3, however the total respondents in roll 4 is 31 and not 30. Can the authors clarify this difference.

6. In lines 35-37, the 30 participants represented 37 professional sectors. Can the authors clarify whether or not some participants represented more than one professional sector. It is also important to know which professional sector (academic institutions, hospitals government agencies and community-based pharmacies) that was represented by the 5 Thai Traditional Doctors.

7. In line 136, the number of academic institutions is given as 21 and in Table 1, roll 5, the number of academic institutions is given as 14. The authors should which of the two figures is correct.

8. Line 169: The authors should revise the sentence.

Reviewer #2:

1. Abstract

The abstract should be organized into clearly defined sections, namely Introduction, Methods, Results, and Conclusion. These components must be distinctly presented within the abstract. At present, the Results section is not adequately delineated, and the principal findings are not clearly articulated.

2. Table 2

a. Please clarify the definitions provided in the “Herbal Medicine Product” section. Specifically, what distinguishes the traditional herbal formula from the health unit herbal formula? Additionally, are household herbal drugs not considered first-line drugs under either category? Kindly explain how household herbal drugs differ from first-line herbal drugs, if they are indeed distinct classifications.

b. Were all the herbal medicine products considered in this research accredited for use by the regulatory body in Thailand?

4. Table 3: With respect to Code 1.5, are the primary care pharmacy products listed under this category classified as herbal medicine products by the NHSO? Please provide clarification in the manuscript.

5. Table 4: The authors are requested to include the following essential information among the listed factors: dosage, active ingredient(s), cautions, batch number, shelf life, and storage conditions.

6. Table 5: The authors are requested to incorporate the following additional factors: knowledge of potential side effects; knowledge of contraindications and specific precautions; knowledge of proper storage conditions; and the ability to provide clear instructions regarding the appropriate use and timing of the product.

7. Table 6: Please include inventory control methods among the listed factors.

8. Discussion: The manuscript requires further careful editing. For example, unnecessary symbols (e.g., dashes) should be removed to improve readability and consistency. Additionally, if AI-assisted tools such as ChatGPT were used in drafting the manuscript, the text should be thoroughly reviewed and edited to ensure clarity, accuracy, and appropriate academic presentation.

9. Please report the means and standard deviations to strengthen the statistical rigor of the study.

Reviewer #3: The english language used must be polished a little; the term "drugstore" is not convenient in this study: rather, the term "Pharmacies" or "herbal medicines facilities" is preferable.

Only descriptive statistics have been used in this manuscript for data analysis which is allowed in such a narrow study.

This study is most beneficial in a country of such demographic characteristics and national terraine. Its very beneficial to the country where the study was conducted using the e-delphi study method.

**********

what does this mean?). If published, this will include your full peer review and any attached files.

If you choose “no”, your identity will remain anonymous but your review may still be made public.

**Do you want your identity to be public for this peer review?**  For information about this choice, including consent withdrawal, please see our Privacy Policy

Reviewer #1: No

Reviewer #2: No

Reviewer #3: **Yes:**  PROFESSOR ISAAC JULIUS ASIEDU-GYEKYE

**********

[NOTE: If reviewer comments were submitted as an attachment file, they will be attached to this email and accessible via the submission site. Please log into your account, locate the manuscript record, and check for the action link "View Attachments". If this link does not appear, there are no attachment files.]

To ensure your figures meet our technical requirements, please review our figure guidelines: https://journals.plos.org/plosone/s/figures

You may also use PLOS’s free figure tool, NAAS, to help you prepare publication quality figures: https://journals.plos.org/plosone/s/figures#loc-tools-for-figure-preparation.

NAAS will assess whether your figures meet our technical requirements by comparing each figure against our figure specifications.

Attachment Submitted filename: Review Comments -PONE-D-25-54073.docx

# Author comment: Author response to Decision Letter 1

**DOI:** 10.1371/journal.pone.0360797.r002

21 Aug 2026

Academic Editor’s Comments: Thank you for submitting your manuscript to PLOS ONE. After careful consideration, we feel that it has merit but does not fully meet PLOS ONE’s publication criteria as it currently stands. Therefore, we invite you to submit a revised version of the manuscript that addresses the points raised during the review process. Aside the uploaded revisions, include a revision of the word "Drug store" in the manuscript

Response: Thank you for this comment. We have revised the terminology throughout the manuscript by replacing “drugstore” and “drugstores” with “pharmacy” and “pharmacies”, respectively. We have also clarified in the Methods that, in the context of this study, the term “pharmacies” encompasses both modern pharmacies and traditional medicine pharmacies operating under the relevant Thai licensing framework.

Journal Requirements: 1. Please ensure that your manuscript meets PLOS ONE's style requirements, including those for file naming.

Response: We have reviewed the revised manuscript and associated submission files and have updated their formatting and file naming, as applicable, to comply with PLOS ONE style requirements.

Journal Requirements: 2. Please provide additional details regarding participant consent. In the ethics statement in the Methods and online submission information, please ensure that you have specified what type you obtained (for instance, written or verbal, and if verbal, how it was documented and witnessed). If your study included minors, state whether you obtained consent from parents or guardians. If the need for consent was waived by the ethics committee, please include this information. Once you have amended this/these statement(s) in the Methods section of the manuscript, please add the same text to the “Ethics Statement” field of the submission form (via “Edit Submission”).

Response: Thank you for this clarification. We have revised the Methods section to explicitly describe the type and documentation of participant consent as follows: “Electronic informed consent was obtained from all participants via the online questionnaire prior to participation. Participants documented their voluntary agreement to participate by checking a consent box before proceeding with the questionnaire.” The same information has been included in the “Ethics Statement” field of the online submission form.

Journal Requirements: 3. Thank you for stating the following financial disclosure: “This study was funded by the Agricultural Research Development Agency (Public Organization), Thailand (Grant No. PRP6505032160).” Please state what role the funders took in the study. If the funders had no role, please state: "The funders had no role in study design, data collection and analysis, decision to publish, or preparation of the manuscript." If this statement is not correct you must amend it as needed. Please include this amended Role of Funder statement in your cover letter; we will change the online submission form on your behalf.

Response: Thank you for this clarification. We confirm that the funders had no role in the study. The following Role of Funder statement has been included in the revised cover letter, as requested: “The funders had no role in study design, data collection and analysis, decision to publish, or preparation of the manuscript.”

Journal Requirements: 4. Thank you for stating the following in the Acknowledgments Section of your manuscript: “TN gratefully acknowledges support from the Royal Golden Jubilee Ph.D. Program (RGJ-Ph.D. Program) under the National Research Council of Thailand, Ministry of Higher Education, Science, Research and Innovation (Grant No. NRCT5-RGJ63001-006).” We note that you have provided funding information that is currently declared in your Funding Statement. However, funding information should not appear in the Acknowledgments section or other areas of your manuscript. We will only publish funding information present in the Funding Statement section of the online submission form. Please remove any funding-related text from the manuscript and let us know how you would like to update your Funding Statement. Currently, your Funding Statement reads as follows: “This study was funded by the Agricultural Research Development Agency (Public Organization), Thailand (Grant No. PRP6505032160).” Please include your amended statements within your cover letter; we will change the online submission form on your behalf.

Response: Thank you for this clarification. We have removed the funding-related statement from the Acknowledgments sections section of the manuscript. We would like to amend the Funding Statement to include both sources of financial support, as follows: “This study was funded by the Agricultural Research Development Agency (Public Organization), Thailand (Grant No. PRP6505032160). TN was supported by the Royal Golden Jubilee Ph.D. Program (RGJ-Ph.D. Program) under the National Research Council of Thailand, Ministry of Higher Education, Science, Research and Innovation (Grant No. NRCT5-RGJ63001-006).” The amended Funding Statement has also been included in the revised cover letter, as requested.

Journal Requirements: 5. We note that your Data Availability Statement is currently as follows: All relevant data are within the manuscript and its Supporting Information files. Please confirm at this time whether or not your submission contains all raw data required to replicate the results of your study. Authors must share the “minimal data set” for their submission.

Response: Thank you for this clarification. We confirm that the revised submission includes the raw data constituting the minimal dataset required to replicate the findings reported in this study. Specifically, the Supporting Information includes de-identified participant characteristics (S1 Dataset); the Round 1 open-ended questionnaire, domain/subcategory mapping, and de-identified original responses (S2 Dataset); and the individual relevance and impact ratings from Rounds 2 and 3 used to calculate the reported descriptive statistics and QL values (S3 Dataset). Accordingly, the Data Availability Statement remains: “All relevant data are within the manuscript and its Supporting Information files.”

Journal Requirements: 6. If the reviewer comments include a recommendation to cite specific previously published works, please review and evaluate these publications to determine whether they are relevant and should be cited. There is no requirement to cite these works unless the editor has indicated otherwise.

Response: We have reviewed the reviewer comments and confirm that no specific previously published works were recommended for citation. Therefore, no additional references were added in response to this requirement.

Journal Requirements: 7. Please review your reference list to ensure that it is complete and correct. If you have cited papers that have been retracted, please include the rationale for doing so in the manuscript text, or remove these references and replace them with relevant current references. Any changes to the reference list should be mentioned in the rebuttal letter that accompanies your revised manuscript. If you need to cite a retracted article, indicate the article’s retracted status in the References list and also include a citation and full reference for the retraction notice.

Response: Thank you for this guidance. We have carefully reviewed the reference list for completeness, bibliographic accuracy, and retraction status. None of the cited articles were identified as retracted. We corrected bibliographic details where necessary and replaced one reference for which the bibliographic information could not be adequately verified with a more appropriate and verifiable source. The reference list has been updated accordingly.

Reviewer #1: The manuscript describes a very important topic that is both relevant and timely as the world shifts gradually back towards natural remedies for disease prevention, treatment and for general wellbeing of the human population. Finding from studies such as this can inform the formulation and rollout of policies to better regulate herbal medicine practice worldwide. There are however some issues with the manuscript that may require the attention of the authors.

Response: We thank the reviewer for the positive assessment of the relevance and potential policy implications of our study. We have carefully considered each of the comments raised and revised the manuscript accordingly, as detailed below.

Reviewer #1: 1. It would have been great to include over the counter medicine sellers (OTCMS) in the study as respondents, since they are the main distributors and dispensers of herbal medicines in most countries.

Response: Thank you for this suggestion. We agree that over-the-counter medicine sellers may represent an important stakeholder group in the distribution and dispensing of herbal medicinal products in some settings. In the present study, however, the expert panel was selected within the Thai healthcare and pharmacy context and included licensed pharmacists and healthcare professionals with relevant experience in managing pharmacies or businesses selling herbal medicinal products. The retail pharmacy sector was represented by six participants. Over-the-counter medicine sellers were not included as a separate distinct stakeholder group. We have acknowledged this limitation in the Discussion and identified broader stakeholder representation as an area for future research: “Additionally, a larger panel, including experts from different professions and sectors, such as over-the-counter medicine sellers, could provide broader perspectives.”

Reviewer #1: 2. Given the importance of the study, a relatively higher number of respondents from a wider professional background would have been great.

Response: Thank you for this comment. We agree that a larger and more professionally diverse expert panel could provide additional perspectives. In this study, 30 experts completed all three rounds of the e-Delphi process. The panel included pharmacists, medical doctors, and Thai traditional doctors from academic institutions, government agencies, hospitals, and pharmacies, with the majority of particpants having more than 10 years of professional experience. The Delphi approach was intended to obtain informed judgements from participants with relevant expertise rather than to achieve statistical representativeness. Nonetheless, we acknowledge that the breath of stakeholder representation could be expanded. We have therefore added this limitation to the Discussion and identified it as an area for future research: “Additionally, a larger panel, including experts from different professions and sectors, such as over-the-counter medicine sellers, could provide broader perspectives.”

Reviewer #1: 3. The there is indication that the questionnaire were administered electronically but the specific software or platform used such google forms etc. was not mentioned

Response: Thank you for pointing out. We have revised the Methods section to specify the electronic platform used to administer the e-Delphi questionnaires. The revised text states: “All three rounds of the e-Delphi questionnaire were administered electronically using Google Forms.”

Reviewer #1: 4. In lines 56, 57, and 63, the abbreviations HPMS and HMPS have used interchangeably. Do they represent the same thing?

Response: Thank you for pointing out this inconsistency. Both “HPMS” and “HMPS” were typographical errors and were intended to refer to herbal medicinal products (HMPs). We have corrected errors and consistently use the abbreviation “HMPs” throughout the revised manuscript.

Reviewer #1: 5. In line 138, Table 1, n=3, however the total respondents in roll 4 is 31 and not 30. Can the authors clarify this difference.

Response: Thank you for identifying this discrepancy. The total number of participants was 30; the error was in the number of pharmacists reported in Table 1. The number of pharmacists was incorrectly reported as 23 and has been corrected to 22. Together with 3 medical doctors and 5 Thai traditional doctors, the corrected total is 30 participants. We have corrected this error in Table 1 has been revised accordingly.

Reviewer #1: 6. In lines 35-37, the 30 participants represented 37 professional sectors. Can the authors clarify whether or not some participants represented more than one professional sector. It is also important to know which professional sector (academic institutions, hospitals government agencies and community-based pharmacies) that was represented by the 5 Thai Traditional Doctors.

Response: Thank you for identifying this discrepancy. The apparent total of 30 affiliations resulted from an error in the number of participants reported as being affiliated with academic institutions. The correct number is 14, not 21. The professional-sector categories were mutually exclusive: academic institutions (n=14), government agencies (n=7), hospitals (n=3), and pharmacies (n=6), giving a total of 30 participants. Regarding the five Thai traditional doctors, all five were affiliated with pharmacies. We have revised the Results section to clarify this point: “All five Thai traditional doctors were affiliated with pharmacies.”

Reviewer #1: 7. In line 136, the number of academic institutions is given as 21 and in Table 1, roll 5, the number of academic institutions is given as 14. The authors should which of the two figures is correct.

Response: Thank you for identifying this inconsistency. The correct number of participants affiliated with academic institutions is 14, as reported in Table 1. The value of 21 in the original narrative text was incorrect and has been corrected to 14 in the revised manuscript.

Reviewer #1: 8. Line 169: The authors should revise the sentence.

Response: Thank you for pointing this out. We have revised the sentence in the Results section to improve its grammatical accuracy and clarity. The revised sentence reads: “The 23 subcategories identified from the open-ended responses in Round 1 were refined and expanded into 34 specific factors, which were categorized into four domains for the closed-ended questionnaire: (1) Herbal medicinal products, (2) Product information, (3) Professionals, and (4) Pharmacy operations.”

Reviewer #2: 1. Abstract. The abstract should be organized into clearly defined sections, namely Introduction, Methods, Results, and Conclusion. These components must be distinctly presented within the abstract. At present, the Results section is not adequately delineated, and the principal findings are not clearly articulated.

Response: Thank you for this comment. We have reorganized the abstract into four clearly defined sections: Introduction, Methods, Results, and Conclusion. We have also revised the Results section to more clearly present the principal findings, including the number of factors that reached consensus, the four identified domains, the factor with the highest consensus score, and the key professional and pharmacy-related factors that reached consensus.

Reviewer #2: 2. Table 2. a. Please clarify the definitions provided in the “Herbal Medicine Product” section. Specifically, what distinguishes the traditional herbal formula from the health unit herbal formula? Additionally, are household herbal drugs not considered first-line drugs under either category? Kindly explain how household herbal drugs differ from first-line herbal drugs, if they are indeed distinct classifications.

Response: Thank you for this comment. We agree that the terminology in Table 2 required clarification. First, “health unit herbal formula” was an inaccurate translation of the Thai term referring to herbal medicines used in public health facilities. We have therefore replaced this term with “Herbal medicines used in public health facilities.” These categories represent different criteria for classifying or selecting herbal medicinal products. A traditional herbal formula is defined according to its basis in traditional medicine knowledge, whereas herbal medicines used in public health facilities are categorized according to their use within the public healthcare system. Similarly, traditional household remedies constitute a regulatory category for household use, whereas first-line herbal medicines are those recommended as initial treatment options for specified conditions. These classifications are therefore not mutually exclusive, and an individual herbal medicinal product may fall within more than one category. To clarify these distinctions, we have added the following note to Table 2: “Traditional household remedies are classified according to their regulatory status for household use, whereas first-line herbal medicines are recommended as initial treatment options for specified conditions. Traditional herbal formulas refer to formulations based on traditional medicine knowledge, while herbal medicines used in public health facilities are categorized according to their use within the public healthcare system. Accordingly, these categories represent different but potentially overlapping criteria for classifying or selecting herbal medicinal products.”

Reviewer #2: 2. Table 2. b. Were all the herbal medicine products considered in this research accredited for use by the regulatory body in Thailand?

Response: Thank you for this question. The regulatory approval status of individual commercial herbal medicinal products was not assessed in this study. Rather, the e-Delphi process identified categories and factors considered relevant to the availability and consumption of herbal medicinal products in Thai pharmacies. Some categories were derived from official Thai regulatory or healthcare frameworks, including the National List of Essential Medicines, traditional household remedies recognized by the Ministry of Public Health, herbal medicines used in public health facilities or under National Health Security Office programs, and Herbal Champions under the National Herbal Action Plan. Other factors, such as widespread use, treatment category, and route of administration, were identified by the expert panel as practical criteria relevant to product selection or classification and do not indicate regulatory approval. Therefore, the categories identified in this study should not be interpreted as indicating that all individual herbal medicinal products were specifically assessed or approved by the Thai regulatory authority.

Reviewer #2: 4. Table 3: With respect to Code 1.5, are the primary care pharmacy products listed under this category classified as herbal medicine products by the NHSO? Please provide clarification in the manuscript.

Response: Thank you for requesting this clarification. Code 1.5 refers to 35 herbal medicinal products included for use in pharmacy-based primary healthcare services under the National Health Security Office (NHSO) program. The NHSO supports the inclusion of these herbal medicines within the specified healthcare service; however, it is not the regulatory authority responsible for the legal classification or approval of herbal medicinal products in Thailand. To avoid implying that the NHSO itself determines their regulatory classification, we have revised Code 1.5 in Table 3 to: “Herbal medicines included for use in pharmacy-based primary healthcare services under the National Health Security Office (NHSO) program”.

Reviewer #2: 5. Table 4: The authors are requested to include the following essential information among the listed factors: dosage, active ingredient(s), cautions, batch number, shelf life, and storage conditions.

Response: Thank you for this suggestion. We agree that dosage, active ingredient(s), cautions, batch number, shelf life, and storage conditions are important elements of herbal medicinal product information. However, these items were not evaluated as separate factors in the closed-ended e-Delphi rounds. We therefore did not add them to Table 4, as doing so retrospectively would imply that they been independently rated by the expert panel and had undergone the predefined consensus assessment. Instead, we have acknowledged these additional product information elements in the Discussion as important considerations for future research. The following sentence has been added: “Future studies should also examine additional product information, such as dosage, active ingredients, cautions, batch number, shelf life, and storage conditions, which were not assessed as separate factors in the closed-ended e-Delphi survey.”

Reviewer #2: 6. Table 5: The authors are requested to incorporate the following additional factors: knowledge of potential side effects; knowledge of contraindications and specific precautions; knowledge of proper storage conditions; and the ability to provide clear instructions regarding the appropriate use and timing of the product.

Response: Thank you for this suggestion. We agree that knowledge of potential adverse effects, contraindications and precautions, proper storage conditions, and the ability to provide clear instructions regarding product use and timing are important professional competerencies of the safe and appropriate use of herbal medicinal products. In the closed-ended e-Delphi rounds, potential adverse effects, contraindications, and precautions were encompassed broadly within the factor “Knowledge about evidence-based efficacy and safety” but were not evaluated as separate factors. Knowledge of proper storage conditions and the ability to provide clear instructions regarding appropriate product use and timing were not independently assessed. We therefore did not add these retrospectively to Table 5, as doing so would imply that they had been independenly rated by the expert panel and subjected to the predefined consensus assessment. Instead, we have acknowledged them in the Discussion as important considerations in future research: “While the present survey addressed knowledge of efficacy and safety broadly, specific professional competencies, including knowledge of potential adverse effects, contraindications, precautions, and proper storage conditions, were not assessed as separate factors. The ability to provide clear instructions on appropriate product use and timing should also be considered in future studies.”

Reviewer #2: 7. Table 6: Please include inventory control methods among the listed factors.

Response: Thank you for this suggestion. Inventory-related issues were raised by experts during the open-ended first round. During content analysis and development of the closed-ended questionnaire, these issues were incorporated into broader themes related to product availability, procurement, and supply chain rather than operationalized as a separate inventory control factor. Consequently, inventory control methods were not independently rated in Rounds 2 and 3.

We therefore did not add inventory control methods retrospectively to Table 6, as doing so would incorrectly imply that this factor had been independently rated by the expert panel and subjected to the predefined consensus assessment. Instead, we have acknowledged this issue in the Discussion and identified it as an area for future research: “Inventory-related issues also emerged during the open-ended round and were considered within the broader themes of product availability, procurement, and supply chain. However, inventory control methods were not independently assessed in the closed-ended e-Delphi survey and should be further examined as a distinct operational factor in future studies.”

Reviewer #2: 8. Discussion: The manuscript requires further careful editing. For example, unnecessary symbols (e.g., dashes) should be removed to improve readability and consistency. Additionally, if AI-assisted tools such as ChatGPT were used in drafting the manuscript, the text should be thoroughly reviewed and edited to ensure clarity, accuracy, and appropriate academic presentation.

Response: Thank you for this comment. We have carefully reviewed and edited the Discussion, as well as the manuscript as a whole, to improve clarity, readability, and consistency. Unnecessary dashes and other stylistic inconsistencies have been removed, and the wording has been revised where appropriate. All revised text has been carefully reviewed by the authors to ensure its accuracy, appropriate academic presentation, and consistency with the study methods, results, and conclusions.

Reviewer #2: 9. Please report the means and standard deviations to strengthen the statistical rigor of the study.

Response: Thank you for this suggestion. We agree that means and standard deviations provide useful complementary information on the distribution of expert ratings. We have therefore calculated the means and standard deviations of the relevance and impact ratings for each factor in Rounds 2 and 3 and included these descriptive statistics in the S3 Dataset. The Methods section has also been revised to describe these analyses. These descriptive statistics complement the QL values, while the predefined QL threshold (≥ 0.75) remains the criterion used to determine consensus in this study. Final consensus status was determined based on the Round 3 QL values.

Reviewer #3: The english language used must be polished a little; the term "drugstore" is not convenient in this study: rather, the term "Pharmacies" or "herbal medicines facilities" is preferable. Only descriptive statistics have been used in this manuscript for data analysis which is allowed in such a narrow study. This study is most beneficial in a country of such demographic characteristics and national terraine. Its very beneficial to the country where the study was conducted using the e-delphi study method.

Response: Thank you for these constructive comments. We have carefully reviewed and edited the manuscript throughout to improve English language, clarity, terminology, and consistency. We have also replaced the term “drugstore” with “pharmacy” or “pharmacies” throughout the manuscript. In the Methods, we further clarified that the term “pharmacies” in this study encompasses both modern pharmacies and traditional medicine pharmacies operating under the relevant Thai licensing framework. We also appreciate the reviewer’s favorable comments regarding the descriptive statistical approach and the relevance of the e-Delphi study to the Thai context.

Attachment Submitted filename: Reponse to Reviewers KP clean.docx

# Editor report: Decision Letter 1

**DOI:** 10.1371/journal.pone.0360797.r003

**Authors:** Ofosua Adi-Dako

2 Oct 2026

Expert consensus on the availability and consumption of herbal medicinal products in Thai pharmacies: An e-Delphi study

PONE-D-25-54073R1

Dear Dr. Pongpirul,

We’re pleased to inform you that your manuscript has been judged scientifically suitable for publication and will be formally accepted for publication once it meets all outstanding technical requirements.

Within one week, you’ll receive an e-mail detailing the required amendments. When these have been addressed, you’ll receive a formal acceptance letter and your manuscript will be scheduled for publication.

An invoice will be generated when your article is formally accepted. Please note, if your institution has a publishing partnership with PLOS and your article meets the relevant criteria, all or part of your publication costs will be covered. Please make sure your user information is up-to-date by logging into Editorial Manager at Editorial Manager® and clicking the ‘Update My Information' link at the top of the page. For questions related to billing, please contact billing support.

If your institution or institutions have a press office, please notify them about your upcoming paper to help maximize its impact. If they’ll be preparing press materials, please inform our press team as soon as possible -- no later than 48 hours after receiving the formal acceptance. Your manuscript will remain under strict press embargo until 2 pm Eastern Time on the date of publication. For more information, please contact onepress@plos.org.

Kind regards,

Ofosua Adi-Dako

Academic Editor

PLOS One

Additional Editor Comments (optional):

Reviewers' comments:

# Editor report: Acceptance letter

**DOI:** 10.1371/journal.pone.0360797.r004

**Authors:** Ofosua Adi-Dako

PONE-D-25-54073R1

PLOS One

Dear Dr. Pongpirul,

I'm pleased to inform you that your manuscript has been deemed suitable for publication in PLOS One. Congratulations! Your manuscript is now being handed over to our production team.

At this stage, our production department will prepare your paper for publication. This includes ensuring the following:

* All references, tables, and figures are properly cited

* All relevant supporting information is included in the manuscript submission,

* There are no issues that prevent the paper from being properly typeset

You will receive further instructions from the production team, including instructions on how to review your proof when it is ready. Please keep in mind that we are working through a large volume of accepted articles, so please give us a few days to review your paper and let you know the next and final steps.

Lastly, if your institution or institutions have a press office, please let them know about your upcoming paper now to help maximize its impact. If they'll be preparing press materials, please inform our press team within the next 48 hours. Your manuscript will remain under strict press embargo until 2 pm Eastern Time on the date of publication. For more information, please contact onepress@plos.org.

You will receive an invoice from PLOS for your publication fee after your manuscript has reached the completed accept phase. If you receive an email requesting payment before acceptance or for any other service, this may be a phishing scheme. Learn how to identify phishing emails and protect your accounts at https://explore.plos.org/phishing.

If we can help with anything else, please email us at customercare@plos.org.

Thank you for submitting your work to PLOS One and supporting open access.

Kind regards,

PLOS One Editorial Office Staff

on behalf of

Dr. Ofosua Adi-Dako

Academic Editor

PLOS One
